# A Robust Assay for Molecular Sexing of Emperor Penguins Using Degraded DNA and the Potential Applicability to Other Spheniscids

**DOI:** 10.1002/ece3.74654

**Published:** 2026-11-05

**Authors:** Vicky Ooi, Sarah R. Smith, Skye Davis, David R. Thompson, Matt H. Pinkerton, Sarah P. Flanagan, Michelle LaRue, Birgitte I. McDonald

**Affiliations:** ^1^ Moss Landing Marine Laboratories San José State University Moss Landing California USA; ^2^ School of Biological Sciences University of Canterbury Christchurch New Zealand; ^3^ New Zealand Institute for Earth Science Wellington New Zealand; ^4^ Gateway Antarctica, School of Earth and Environment University of Canterbury Christchurch New Zealand

**Keywords:** amplification refractory mutation system (ARMS), degraded DNA, non‐invasive sampling, seabirds, sex‐linked marker, Sphenisciformes

## Abstract

Molecular sexing is a widely used approach for identifying the sex of monomorphic birds, but existing primers often target relatively large products that are difficult to amplify from highly degraded DNA. In this study, we developed a new primer set (voEP_F/voEP_R/P2) based on allele‐specific polymerase chain reaction (ASPCR) to facilitate the sexing of emperor penguins ( *Aptenodytes forsteri* ) using non‐invasively collected guano. Our assay targets short sequences within a conserved exon of the chromodomain helicase DNA binding 1 (*CHD1*) gene and can therefore be used with a broad range of sample types. We compared the performance of our new primer set in both plucked feathers and guano to the commonly used intron‐based sexing primers (2550F/2718R and P8/P2) and evaluated its sensitivity through a cross‐factorial fragmentation and dilution experiment of synthetic *CHD1* templates. We then independently validated the accuracy of our assay in a small subset of feather samples using whole‐genome sequencing (WGS) data. Although P8/P2 was highly effective compared to 2550F/2718R for sex determination in emperor penguins, it was less reliable when used with guano samples. In contrast, the voEP primers consistently yielded higher amplification success and showed greater analytical sensitivity across both feathers and guano. Sequence alignments of *CHD1* from multiple penguin species revealed conservation of the primer‐binding sites, suggesting that the newly designed primers can potentially be used with other Spheniscids. Our results demonstrate that the voEP assay is a robust and reliable alternative method for sex identification in penguins and may provide a solution for sexing with lower‐quality genetic samples.

## Graphical abstract

Molecular sexing of birds with intron‐based primers often targets relatively large amplicons that are difficult to amplify with degraded DNA. In this study, we developed a new primer set based on allele‐specific PCR to facilitate sexing of emperor penguins (*Aptenodytes forsteri*) using non‐invasively collected guano. By targeting short sequences within a conserved exon of the *CHD1* gene, our assay remains effective across a broad range of sample types and may also be used for sex assignments in other penguin species.

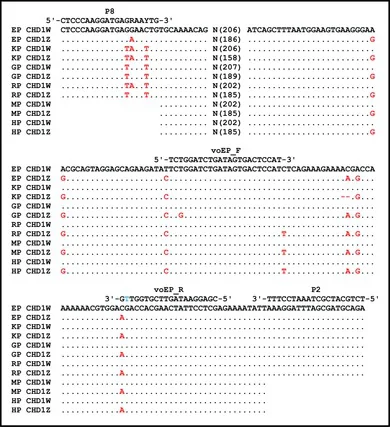

## 1 Introduction

Accurate identification of sex in avian wildlife is essential to the investigation of population structure, behavioral ecology, and evolution (Morinha et al. 2012). However, approximately half of all bird species are sexually monomorphic, making their sex difficult to determine based on external morphology (Griffiths 2000). Molecular methods that identify variations between avian sex chromosomes (W and Z) are thus widely used for the sexing of monomorphic birds (Dubiec and Zagalska‐Neubauer 2006; Morinha et al. 2012; Tagliavia et al. 2023). Although various primer sets have been developed for avian sexing, many rely on amplification of relatively large fragments that are ~300 bp or more (Griffiths et al. 1998; Fridolfsson and Ellegren 1999; Ito et al. 2003; Lee et al. 2010), which limits their use with degraded DNA samples (Lee and Griffiths 2003; Sefc et al. 2003; Bantock et al. 2008).

The standard approach of molecular sexing in non‐ratite birds amplifies across the intron of the sex‐linked chromodomain helicase DNA binding 1 (*CHD1*) gene (Griffiths et al. 1998; Kahn et al. 1998; Fridolfsson and Ellegren 1999). This gene is highly conserved between species, but some variations exist in the length and nucleotide sequence between the intronic regions of *CHD1W* and *CHD1Z* (Griffiths et al. 1998; Morinha et al. 2012). Due to the intron length difference, two products of different sizes are typically obtained for heterogametic females (WZ), while only one product is obtained for homogametic males (ZZ). Since entire introns are amplified, relatively large amplicons are generated. As a result, this form of sexing becomes unreliable when highly degraded or fragmented DNA samples are used (Lee and Griffiths 2003; Bantock et al. 2008), as amplification failure of one (i.e., allelic dropout) or both alleles (i.e., locus dropout) may lead to misidentification of sex or the inability to sex‐type individuals (Arnold et al. 2003; Bantock et al. 2008; Tvedebrink et al. 2012).

Allele‐specific polymerase chain reaction (ASPCR) offers an alternative approach for molecular sexing in birds (Ito et al. 2003; He et al. 2005; Lee et al. 2008). This method uses a specific primer design to selectively amplify an allele of interest (Wu et al. 1989; Newton et al. 1989). As *Taq* DNA polymerase lacks a 3′ to 5′ exonuclease activity, a terminal mismatch at the 3′ end of primer‐template duplexes can significantly reduce amplification efficiency (Ugozzoli and Wallace 1991). Consequently, if an allele‐specific primer is designed to selectively pair with the nucleotide form that occurs only on *CHD1W*, then a PCR product will only be obtained in females (Ito et al. 2003; He et al. 2005). Simultaneously, a pair of primers must be synthesized to amplify a common product in both sexes so that a successful PCR can be verified (Ito et al. 2003). Although this technique has been used for avian sexing, existing primers still target intronic regions, which yield similarly large amplicon fragments (~300–400 bp) (Ito et al. 2003; He et al. 2005; Lee et al. 2008). Since ASPCR‐based sexing does not require amplification across a fixed region, such as an intron, smaller products can be targeted, particularly in conserved exons.

Antarctic penguins are organisms for which an ASPCR‐based approach to sexing would be ideal. Not only are most species plumage monomorphic (Loureiro et al. 2014; Polito et al. 2024), but they also reside in remote areas where human access is limited. As a result, tissue samples that contain high quality and quantity of DNA (e.g., biopsies, plucked feathers, blood) are logistically more difficult to obtain. Collecting such samples also requires the capture and handling of individuals (Hart et al. 2009; Valenzuela‐Guerra et al. 2013), which may limit sample sizes because field seasons are generally short in these locations. This limitation is accentuated when larger species, such as emperor penguins ( *Aptenodytes forsteri* ), are targeted as more effort is required to restrain and sample them (Cockrem et al. 2008; Houstin et al. 2022). Although non‐invasively collected guano may be a preferred source of genetic material for these animals (Xavier et al. 2018; Tian et al. 2021), it yields DNA of lower quality and quantity that may be challenging to amplify (Taberlet et al. 1999; Ooi et al. 2023). Having a sexing assay that can be reliably used with degraded DNA is therefore highly valuable for the research and monitoring of these penguins.

Here, we developed a new primer set (voEP_F/voEP_R/P2) based on ASPCR that targets short regions within the conserved *CHD1* exons to facilitate sexing of emperor penguins using non‐invasively collected guano. We evaluated the efficacy of our new primer set in both plucked feathers and guano by comparing its performance to the commonly used intron‐based sexing primers (2550F/2718R and P8/P2). We also assessed its sensitivity by systematically fragmenting and diluting synthesized *CHD1* genes, which allowed us to isolate the effects of degradation and variable amounts of template DNA. To validate the accuracy and reliability of our assay, we compared the sexes determined in a small subset of samples to the independent results yielded via whole‐genome sequencing (WGS). Finally, we aligned the newly developed primers against existing *CHD1* sequences of king ( *Aptenodytes patagonicus* ), gentoo ( *Pygoscelis papua* ), southern rockhopper ( *Eudyptes chrysocome* ), Magellanic ( *Spheniscus magellanicus* ), and Humboldt ( *Spheniscus humboldti* ) penguins to assess their potential applicability to other Spheniscidae species.

## 2 Materials and Methods

### 2.1 Sample Collection

Dorsal contour feathers were obtained from emperor penguins of unknown sex at Cape Crozier in November 2022 (*n* = 31) and 2024 (*n* = 29) and north of Marie Byrd Land in February 2023 (*n* = 34). Feathers were plucked from penguins that were captured as part of a different biologging study. The calamus (~0.4 cm in length) was trimmed from each plucked feather and stored in a 2 mL cryovial filled with 95% or 99% ethanol. Additional feathers were also obtained from 18 of the captured individuals and sent to the University of Canterbury in New Zealand for sex validation using whole‐genome sequencing (WGS). Guano samples were opportunistically collected in November 2022 (*n* = 60) and 2024 (*n* = 109) from emperor penguins that were observed to defecate at the edge of the Cape Crozier colony. These samples were collected by two independent researchers stationed at different sections of the colony to minimize the risk of duplicate sampling. Any guano samples collected within a short distance of one another were observed to originate from different penguins. Given that the penguins continually arrived and departed the colony within 24 h, the probability of repeated sampling from the same individual was relatively low. A disposable spatula was used to fill approximately one‐third of a 2 or 5 mL cryovial with freshly eliminated guano, and the remaining volume was filled with 95% or 99% ethanol. All feather and guano samples collected were stored within a cooler at −5°C to −15°C in the field and subsequently kept frozen at −80°C until further processing.

### 2.2 DNA Extraction

#### 2.2.1 Feather

Each calamus was removed from ethanol and rinsed with 500 μL of nuclease‐free water prior to DNA extraction. The calamus was minced into small pieces with a sterile razor blade and placed in a 1.5 mL microcentrifuge tube. DNA extraction was initially tested with the DNeasy Blood and Tissue kit (#69504, Qiagen, Germany), but low yields of DNA were retrieved. Therefore, subsequent DNA isolations were performed using a slightly modified version of the InstaGene isolation protocol (Freedman et al. 2008). Briefly, 200 μL of InstaGene matrix (Bio Rad), 3 μL of 20 mg/mL Proteinase K (Qiagen), and 9 μL of 1 M dithiothreitol (DTT) were added to the sample for cell lysis. After a brief vortex, the mixture was pulse‐centrifuged and left to incubate at 65°C overnight in a VorTemp 56 shaking incubator (Labnet International, USA) set at 1000 rpm. The sample was centrifuged at 9300 *g* for 30 s and incubated at 110°C in a heat block for 10 min. Following incubation, the sample was vortexed, centrifuged at 13,200 *g* for 3 min, and the supernatant was transferred into a new 1.5 mL microcentrifuge tube. The centrifugation process was repeated once more, and the supernatant was collected as final isolate.

#### 2.2.2 Guano

Each guano sample was centrifuged for 5 min at 5000 *g* and ethanol was poured out before DNA extraction. Approximately 150–200 mg of pelleted guano was transferred into a new 2 mL microcentrifuge tube and washed four to five times with 1 mL of phosphate‐buffered saline (PBS). During each wash, the sample mixture was briefly vortexed to resuspend the guano, centrifuged to re‐pellet the suspended particles, and the supernatant was discarded. DNA was then extracted from *ca*. 65 mg of washed guano using the QIAamp PowerFecal Pro DNA kit (#51804, Qiagen, Germany) with three modifications to the manufacturer's protocol. Firstly, after the addition of Solution CD1, the mixture was incubated at 65°C for 10 min and homogenized with a Mini‐BeadBeater‐16 (BioSpec Products, USA) for two 30 s cycles with a 30 s interval. Secondly, the wash with 500 μL of Solution C5 was repeated four to five times or until the silica membrane became translucent or colorless. Lastly, 50 μL of Solution C6, pre‐warmed to 56°C, was added to the silica membrane and incubated at room temperature for 5 min before centrifugation to elute the DNA.

### 2.3 DNA Quantification

Using 2 μL of DNA extract, the concentration (ng/μL) and purity (A260/280 and A260/230) of the isolated DNA were quantified on a NanoDrop 1000 Spectrophotometer (Thermo Fisher Scientific, USA). All DNA extracts were stored at −20°C (short‐term) or −80°C (long‐term) until further analysis.

### 2.4 PCR and Gel Electrophoresis

PCR was conducted with an Arktik Thermal Cycler (Thermo Fisher Scientific, Finland) using a 25 μL reaction volume, each consisting of 1× GoTaq Green Master Mix (#M712, Promega), 0.2 μM of corresponding forward and reverse primers for each primer set (Table 1), an additional 0.5 mM of MgCl_2_, approximately 2–350 ng (feathers) or 1–200 ng (guano) of genomic DNA, and nuclease‐free water. While the amount of DNA yielded from different samples was highly variable, all reactions were performed using the same template quantity for each sample to ensure comparable results across the primer sets. A no‐template control, where DNA was replaced with nuclease‐free water, was used to detect contamination for all PCRs.

**TABLE 1:** Forward and reverse primers used in this study with their corresponding amplicon sizes.

Primer set	Forward primer	Reverse primer	Amplicon size (bp)
2550F/2718R	2550F (Fridolfsson and Ellegren 1999)	2718R (Fridolfsson and Ellegren 1999)	W: ~750 Z: ~650
P8/P2	P8 (Griffiths et al. 1998)	P2 (Griffiths et al. 1998)	W: 388 Z: 368
voEP	voEP_F (This study)	voEP_R (This study) P2 (Griffiths et al. 1998)	W: 74 Z and W: 104

*Note:* The amplicon sizes of P8/P2 and voEP primers for emperor penguins were determined in this study, while the expected amplicon sizes of 2550F/2718R were inferred from other penguin species (*cf*. Zhang et al. 2013).

Thermal cycling conditions for the 2550F/2718R and P8/P2 primers were 94°C for 2 min, 35–40 cycles of 94°C for 30 s, 50°C for 45 s, and 72°C for 45 s, with a final extension at 72°C for 5 min. The cycling conditions for the voEP primer set were 95°C for 3 min, followed by 35–40 cycles of 95°C for 30 s, 56.2°C for 45 s, and 72°C for 20 s, with a final extension at 72°C for 5 min. All samples were amplified with 35 PCR cycles, but the number of cycles was raised incrementally up to 40 if faint bands were yielded. PCR products were initially separated by electrophoresis on a 3% ethidium bromide (EtBr)‐stained agarose gel in tris‐acetate‐EDTA (TAE) buffer for 1 h 30 min (2550F/2718R and voEP) or 1 h 45 min (P8/P2) at 10 V/cm. However, the band separation was limited for P8/P2 primers. Electrophoresis was subsequently performed with a 3% EtBr‐stained agarose gel in lithium acetate‐borate (LAB) buffer for 45 min (voEP) or 1 h 45 min (P8/P2) at 20 V/cm. The resulting bands, visualized with a UV transilluminator, were interpreted blindly to determine the sex of the emperor penguins. Gel images were converted to 16‐bit grayscale format, and the ‘brightness/contrast’ or ‘window/level’ were adjusted for the entire gel using the ImageJ software (version 1.8.0, National Institutes of Health) to improve visualization.

As the primer sets used in this study either targeted distinct regions of the *CHD1* gene (2550F/2718R vs. P8/P2) or relied on different molecular principles for sex determination (intron size vs. sex‐specific allele), there is only a small probability that different assays would independently produce the same incorrect sex assignment due to PCR artifacts. Therefore, matching sexes yielded by different assays were used as internal validation for the sex determined. To further ensure the accuracy of sexing results, PCR was repeated two to three times in samples that yielded faint/ambiguous (i.e., not clearly identifiable) bands or when different primers produced discordant results. The final sex assignment was then determined based on consensus. However, samples were assigned as males when different replicates yielded conflicting sexes to highlight allelic dropout for statistical comparisons. Sample‐level details for the PCR replicates are provided in the dataset available at https://github.com/vicky‐ooi/EP_Molecular_Sexing.

### 2.5 Sequencing of CHD1 Alleles and New Primer Design

PCR products amplified using the P8/P2 primers from a male and female emperor penguin were run on a 3% agarose gel at 10 V/cm for an extended period (~3 h) to obtain good separation of the *CHD1W* and *CHD1Z* bands. Both band fragments were excised from the agarose gel with a razor blade and purified using the Monarch Spin DNA Gel Extraction kit (#T1120S, New England Biolabs). Purified PCR products were quantified and quality‐checked on a NanoDrop 1000 Spectrophotometer (Thermo Fisher Scientific, USA) prior to being Sanger sequenced in the forward and reverse directions (Eurofins Genomics). Raw sequences were imported into Geneious Prime 2024.0.7 and low‐quality ends were trimmed from the reads. The trimmed forward and reverse reads were aligned to generate a consensus sequence for each PCR product (Figure 1). The *CHD1* sequences were then deposited in Genbank under the accession numbers PV563863 and PV563864.

**FIGURE 1:**
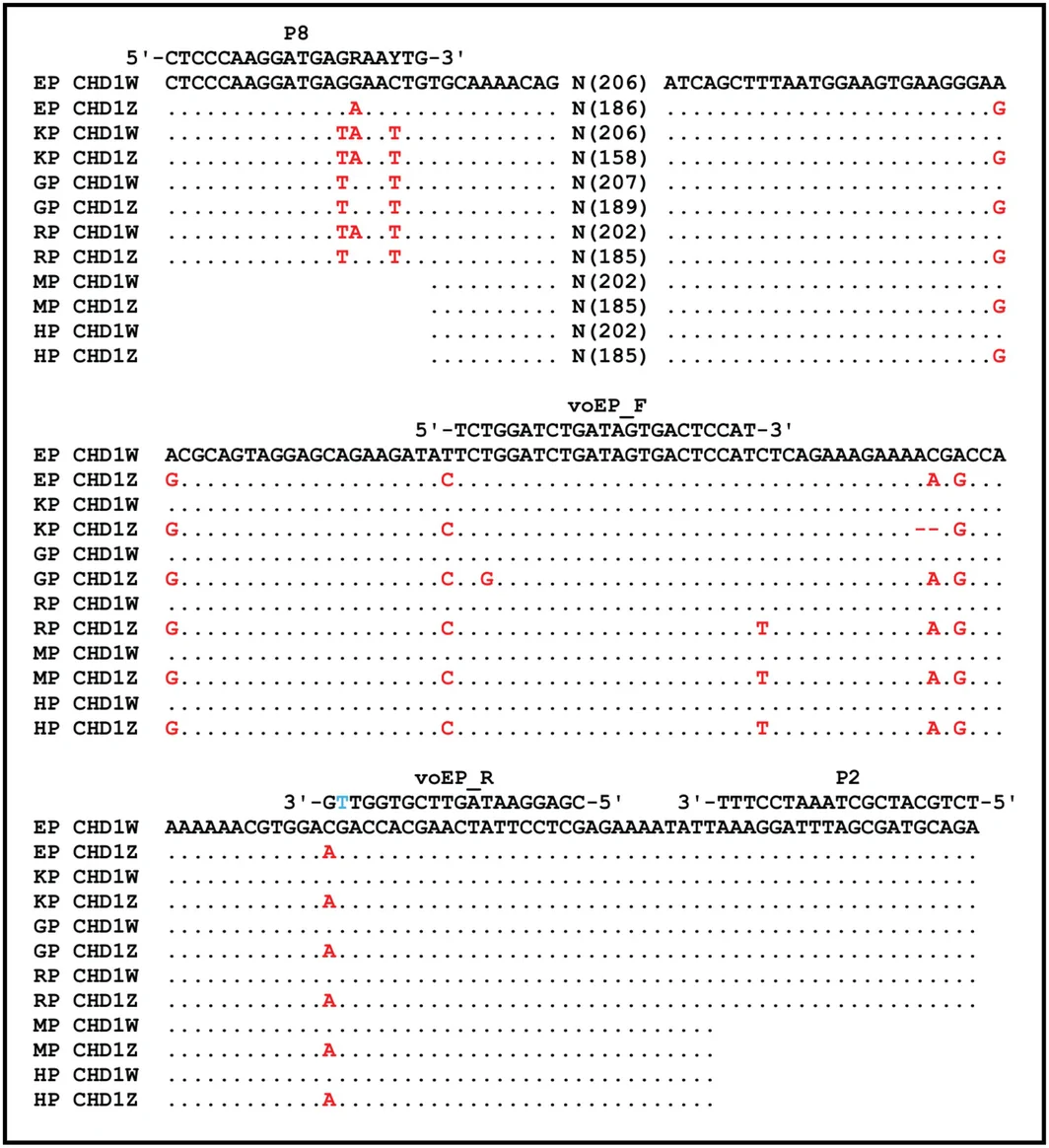
Alignment of the *CHD1W* and *CHD1Z* exonic sequences from six penguin species, and the locations of primer binding sites. Conserved residues are indicated with dots, sequence variants are shown in red, and unknown sequences are indicated with open spaces. Intron location is denoted as N (intron length in bp). The blue nucleotide (voEP_R primer) represents the additional mismatch added to destabilize the primer‐template duplexes. EP, emperor penguin; GP, gentoo penguin; HP, Humboldt penguin; KP, king penguin; MP, Magellanic penguin; RP, southern rockhopper penguin.

The *CHD1* sequences obtained from emperor penguins were used to develop the voEP_F and voEP_R primers in SnapGene (version 7.2.1) (Figure 1). The allele‐specific primer (voEP_R) was designed with a mismatch at the 3′ end to the *CHD1Z* sequence to enable the amplification of a female‐specific product. Since the 3′ terminal mismatch (G/A) has a weak destabilizing strength, a strong destabilizing mismatch (T/G) was added at the penultimate base to increase the primer specificity (Little 1995; Liu et al. 2012). The voEP_F and P2 primers were designed to amplify a common product in both *CHD1W* and *CHD1Z* (Figure 1). Analyses of primer dimers and secondary structures for the newly designed voEP_F/voEP_R/P2 assay were conducted using the Integrated DNA Technologies (IDT) OligoAnalyzer tool.

### 2.6 Comparative Sensitivity Analysis With Synthetic CHD1 Templates

Since an annotated *CHD1* gene sequence for emperor penguins is not available, the partial 368 bp *CHD1Z* sequence obtained in this study was blasted against the only available male emperor penguin reference genome (GCA_000699145.1) on Genbank. Using the matching region as a central sequence, 1066 bp of upstream and downstream flanking sequences were extracted from the subject (Accession number: KL225609.1) to yield a 2500 bp *CHD1Z* fragment. The same flanking sequences (1056 bp upstream and downstream) were also added to the 388 bp *CHD1W* sequence to produce a corresponding 2500 bp *CHD1W* fragment. The flanking sequences were then optimized with the IDT codon optimization tool to reduce the complexity for final synthesis by Twist Bioscience; the primer‐binding region and sequences in between were left unmodified (Table S1). The synthesized *CHD1Z* was used to represent a male emperor penguin, while *CHD1Z* and CHD1*W* mixed at a 1:1 ratio were used to represent a female.

DNA stocks for male (ZZ) and female (ZW) were prepared at a concentration of 0.2 ng/μL. To simulate DNA degradation, four aliquots of the 2500 bp stocks were sheared to average sizes of 1000, 600, 300, and 150 bp, respectively, using the M220 focused‐ultrasonicator (Covaris, USA). To determine the effects of template quantity, DNA at each fragment size was serially diluted to concentrations of 2 × 10^−3^, 2 × 10^−4^, 2 × 10^−5^, 2 × 10^−6^, and 2 × 10^−7^ ng/μL. The average fragment sizes and DNA concentrations were subsequently verified using an Agilent 2100 Bioanalyzer. A crossed factorial amplification experiment was then conducted with the P8/P2 and voEP primer sets using 1 μL of DNA, following the optimized protocols with 35 PCR cycles. PCR was repeated an additional time on a different day for any experimental condition that yielded hazy bands and/or failed to show a clear amplification trend, and the run demonstrating the clearest trend was selected for visual presentation.

### 2.7 Independent Sex Validation With Whole‐Genome Sequencing (WGS)

Sex validation was conducted by the Flanagan Lab at the University of Canterbury in New Zealand. DNA was extracted from 18 feather samples with the Qiagen DNeasy Blood and Tissue kit (#69504, Qiagen, Germany). The spin‐column protocol for the purification of total DNA from animal tissues was conducted with several modifications to maximize DNA yield: (1) the calamus of each feather was chopped finely and digested overnight with 20 μL of proteinase K (Qiagen) and 10 μL of 10 M DTT (ThermoFisher Scientific) at 56°C, followed by an additional 3 h digest with 20 μL of proteinase K on the following day, (2) the eluate was passed through the spin column twice during the DNA binding step, and (3) two final 100 μL elution steps were used, with AE buffer pre‐heated to 70°C and samples incubated at room temperature for 5 min prior to centrifuging. DNA samples were purified and concentrated to a 30 μL total volume using Mag‐Bind TotalPure NGS magnetic beads (Omega Bio‐Tek). DNA yield and quality were checked with agarose gel electrophoresis and the Qubit Broad Range assay kit (ThermoFisher Scientific). Seven samples met the minimum DNA yield requirements for WGS and were dried in DNA stabilization tubes (GenTegra) at room temperature for 30 min in a Savant SPD131DDA SpeedVac Concentrator (ThermoFisher Scientific). Samples were sent to GENEWIZ (Azenta Life Sciences) for medium coverage (≈20×) WGS. Each library was prepared using the VAHTS Universal Plus DNA Library Prep Kit for Illumina V2 (Vazyme) with PCR enrichment, and pooled based on their effective concentration as determined by Qubit 3.0 and the Agilent 2100 Bioanalyzer System. Sequencing was then performed across multiple paired‐end 150 bp NovaSeq X Plus 25B lanes (Illumina) alongside other emperor penguin samples.

Raw reads were processed using fastp v1.3.3 (Chen 2025) to automatically detect and remove adapter sequences, retain reads with Phred ≥ 15 and read length ≥ 15 bp. FastQC v0.12.1 was used to assess the quality of sequence data. As a reference genome with both Z and W chromosomes was unavailable for emperor penguins, the processed paired‐end reads were mapped with bwa‐mem2 v2.3 (Vasimuddin et al. 2019) to a female king penguin chromosome‐level reference genome (NCBI RefSeq accession GCF_965638725.1). Samtools v1.23.1 (Danecek et al. 2021) and picard v3.4.0 were used to sort and index BAM files and mark PCR and optical duplicates. Sex inference was performed with the package XYalign v.1.1.5 (Webster et al. 2019) using the ‘CHARACTERIZE_SEX_CHROMS’ and ‘CHROM_STATS’ functions with default settings. Total read counts, average read depth, and average mapping quality were calculated for the Z and W chromosomes and standardized using an autosome similar in size to the Z (chromosome 4). Ratios of these metrics were used to cluster samples into males and females, as males (ZZ) were expected to have relatively fewer reads mapping confidently to the W chromosome compared to females (ZW). This analysis was conducted twice, once without the default XYalign quality filters (‘processed’) and once with the filters (‘pre‐processed’), to determine whether data processing impacts sex identification. The sexes inferred via WGS in these samples were then manually compared to those determined with the P8/P2 and voEP primers to validate the accuracy and reliability of those assays.

### 2.8 Sequence Alignments

*CHD1W* and *CHD1Z* sequences of king, gentoo, and southern rockhopper penguins were obtained from Zhang et al. (2013), and those of Magellanic and Humboldt penguins were retrieved from Hibbets et al. (2020). The introns of all sequences were removed, and the exons were aligned against the emperor penguin *CHD1W* sequence using the NCBI Basic Local Alignment Search Tool (BLAST). The P8/P2 and voEP primers were then manually aligned against the *CHD1* sequences of the six Spheniscidae species (Figure 1). This alignment was used to assess the potential applicability of the voEP primers to other penguins.

### 2.9 Statistical Analysis

Paired statistical tests were used to compare the performance of P8/P2 and voEP primers in R v.4.5.0 (R Core Team 2025) using the significance threshold of *α* = 0.05. The number of samples that failed to amplify with each assay was compared using exact McNemar's test from the *exact2x2* package (Fay 2010) for each sample type. This was used to evaluate whether the amplification rate was better with one primer set than the other for feathers and guano. For samples that amplified with both assays, the degree of concurrence between both primers was calculated using the Cohen's Kappa (κ) coefficient via the *psych* package (Revelle 2026). This was used to estimate the reliability of the voEP assay compared to previously established P8/P2 primers. The discordant sex assignments yielded by both primer sets were then compared using exact McNemar's test (*exact2x2* package; Fay 2010) to determine whether they showed a directional bias (i.e., one assay consistently produced a particular sex). The dataset and analytical codes used are available at https://github.com/vicky‐ooi/EP_Molecular_Sexing.

## 3 Results

### 3.1 Amplification of CHD1W and CHD1Z With All Primer Sets

The 2550F/2718R primer set was tested with 40 feather samples. Although 37 out of 40 yielded PCR products within the expected size range, many showed faint bands that were ambiguous for sexing (Figure 2A and Figure S1). The use of a higher MgCl_2_ concentration (2 mM vs. 1.5 mM) increased the band intensity, enabling detection in more samples, but off‐target amplification became more prevalent (Figure 2A and Figure S1). Different annealing temperatures and cycle numbers were also tested to optimize the PCR; however, no significant improvement was achieved (results not shown). Of the samples that amplified successfully with this primer set, five yielded only one band despite being detected as females with both the P8/P2 and voEP primers (Figures S1–S3). Since allelic and locus dropouts were prevalent, even in feather samples with higher DNA yields, this primer set was not used for further comparisons.

**FIGURE 2:**
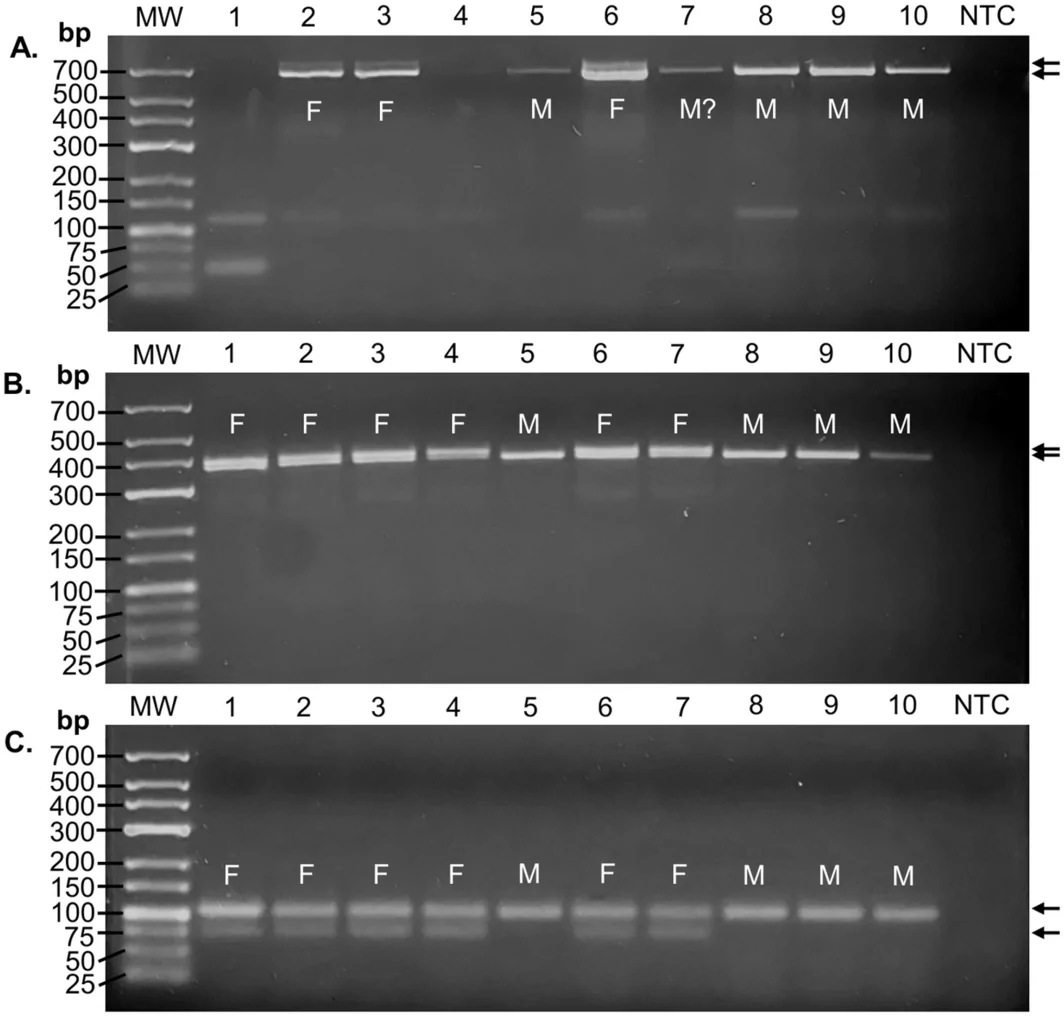
Electrophoretic band patterns of PCR products yielded with 3% agarose gel in TAE buffer for the representative emperor penguin samples. The figure shows results from PCR assays using the (A) 2550F/2718R primers, (B) P8/P2 primers, and (C) voEP_F/voEP_R/P2 primers. Arrows on the right indicate the location and fragment size of the expected/actual bands for male (M) and female (F) emperor penguins in each assay. The gels shown here correspond to those in Figures S1A, S2A, and S3A. MW, molecular weight ladder; NTC, no‐template control.

Expected band patterns for males and females were obtained with both the P8/P2 and voEP primer sets (Figure 2B,C). Amplification success rates were similar between both assays in the feather samples (P8/P2 = 96.8%, voEP = 100%; Exact McNemar's test: odds ratio (OR) = 0, 95% confidence interval (CI) = 0–2.42, *p* = 0.25; Figure 3A). However, the voEP primers successfully amplified significantly more samples compared to P8/P2 when guano samples were analyzed (P8/P2 = 88.8%, voEP = 93.5%; Exact McNemar's test: OR = 0, 95% CI = 0–0.28, *p* = 6.10 × 10^−5^; Figure 3A).

**FIGURE 3:**
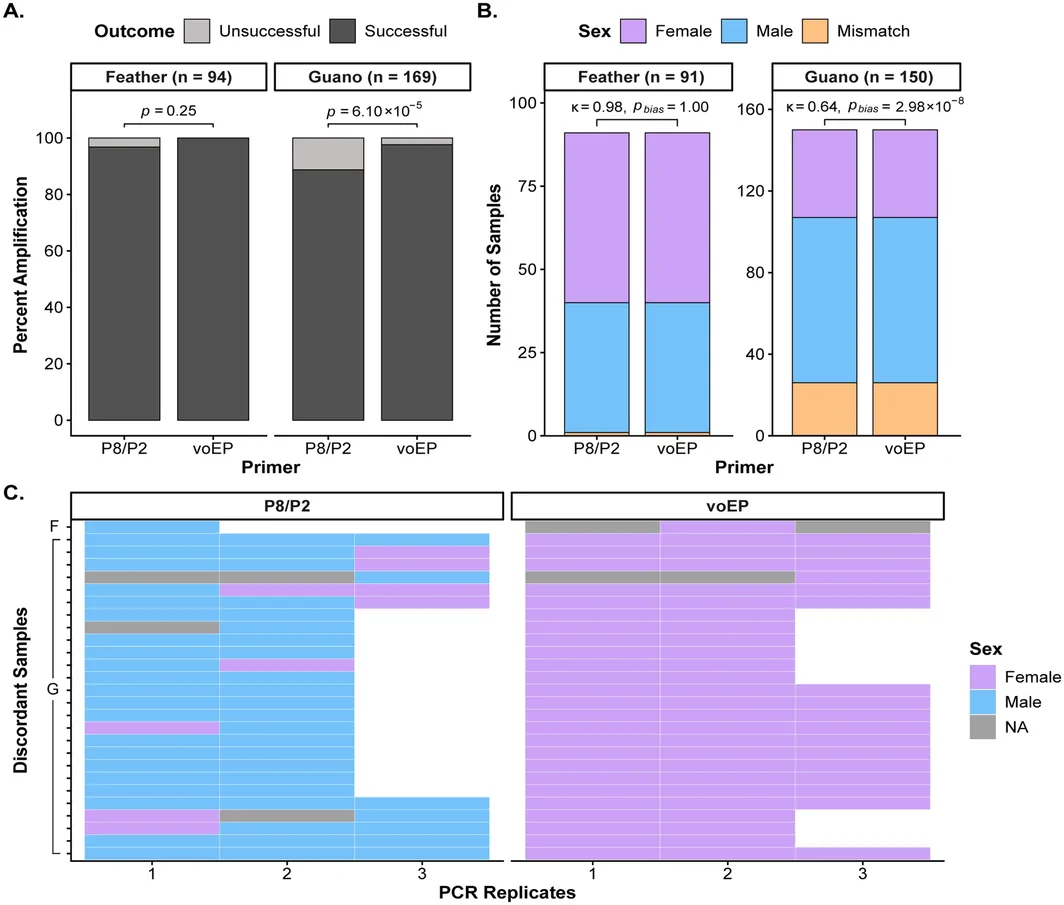
A summary of sexing results yielded with P8/P2 and voEP primer sets. (A) Percentage of successful and unsuccessful amplifications in feathers and guano. (B) The number of samples that showed concordant and discordant results in both sample types. Cohen's Kappa (κ) revealed an almost perfect agreement between both primer sets in feather samples. The mismatches observed in guano samples showed a significant directional bias. (C) A closer examination on the PCR replicates of discordant samples (F = feather, G = guano) revealed allelic dropouts in 8 guano samples when the P8/P2 assay was used. NA represents no amplification yielded in the replicate.

Sexing results agreed almost entirely between both primer sets for the feather samples, with only one mismatch observed (Cohen's κ = 0.98, 95% CI = 0.93–1.00, *n* = 91; Exact McNemar's test: OR = 0, 95% CI = 0–39, *p*_*bias*_ = 1.00; Figure 3B), which was suspected to be due to a cross‐contamination event. For sex determination using guano, however, the level of agreement between both primer sets was substantial but lower than that observed with the feather samples (Cohen's κ = 0.64, 95% CI = 0.52–0.76, *n* = 150; Figure 3B). A significant unidirectional bias was detected between the two assays across the 26 discordant guano samples (Exact McNemar's test: OR = 0, 95% CI = 0–0.15, *p*_*bias*_ = 2.98 × 10^−8^). In all mismatched cases, the P8/P2 primers identified the samples as males (one band), while the voEP primers identified the samples as females (two bands) (Figures S2–S5). In 30.8% (8/26) of the discordant guano samples, at least one PCR replicate for the P8/P2 primer set yielded a female assignment, which suggests the occurrence of allelic dropout (Figure 3C).

Most of the samples that showed a single band were assigned as males. However, two samples yielded a single band, by each the P8/P2 and voEP primers, that corresponded to the female‐specific fragment and were thus identified as females. Occasionally, faint secondary PCR products were obtained by both primer sets in samples that contained low quantity and quality of DNA; these did not affect sex assignment as they were not of the expected product sizes. Notably, the resolution between the bands produced was highly limited with the P8/P2 primers when a standard agarose gel in TAE buffer was used (Figure 2B). Using a less conductive LAB gel allowed twice the voltage to be applied without generating excessive heat. Under this condition, the electrophoresis time for voEP primers was reduced by half, while a slightly larger band separation was achieved over the same run time for P8/P2 primers (Figure 4A).

**FIGURE 4:**
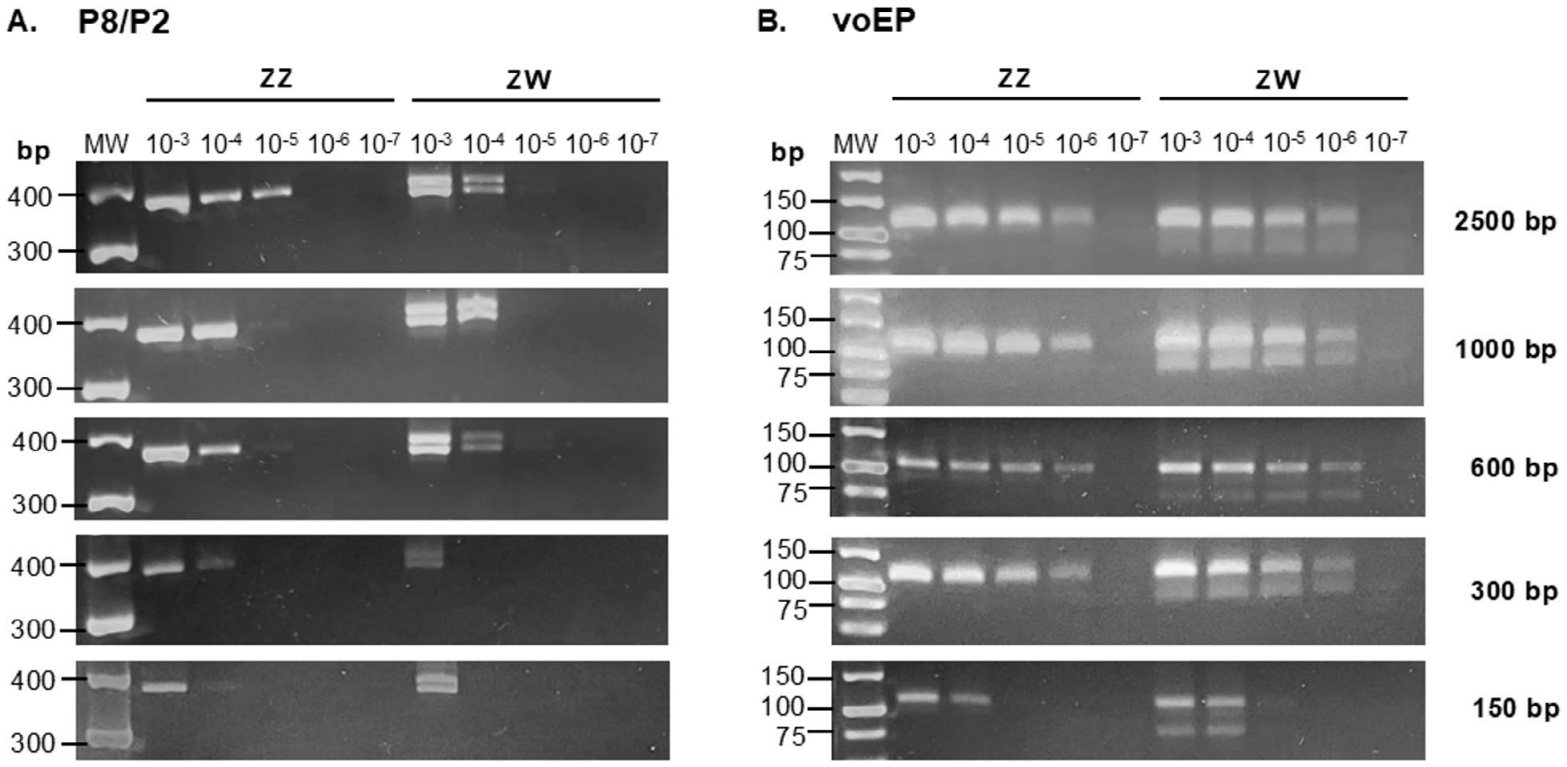
Primer sensitivity analysis via fragmentation and dilution of synthetic *CHD1* templates. Amplification results of ZZ (male) and ZW (female) across different template quantities (2 × 10^−3^, 2 × 10^−4^, 2 × 10^−5^, 2 × 10^−6^, 2 × 10^−7^ ng) and average template DNA sizes (2500, 1000, 600, 300, 150 bp) yielded with (A) P8/P2 and (B) voEP primer sets on 3% lithium acetate‐borate (LAB) gels.

### 3.2 Primer Sensitivity to Degradation and Quantity of Input DNA

Both P8/P2 and voEP primer sets consistently yielded one band for male (ZZ) and two bands for female (ZW) (Figure 4A,B). At average fragment sizes of 2500, 1000, 600, and 300 bp, the voEP primers produced strong and clearly distinguishable bands for both male and female down to a template DNA quantity of 2 × 10^−6^ ng. At an average fragment size of 150 bp, the voEP primer set produced strong and distinct bands for both sexes down to a template DNA quantity of 2 × 10^−4^ ng. In comparison, the P8/P2 primers required 10‐ to 100‐fold more DNA at larger fragment sizes (2500–600 bp) and up to 1000‐fold more DNA at smaller fragment sizes (300–150 bp) to yield robust and clearly distinguishable bands for sexing (Figure 4).

### 3.3 Independent Sex Validation Using WGS

The sexes of the seven samples inferred via WGS corroborated their consensus sex assignments using both P8/P2 and voEP primer sets (Table 2, Figure S6). As expected, the chrZ/chr4 read depth ratios were ≈0.5 for the six females (ZW) and ≈1.0 for the single male identified (ZZ), regardless of whether pre‐processed or processed data (i.e., additional variant quality filters) were used (Table S2). However, pre‐processed data enabled better delineation of the sexes based on chrW/chr4 read depth ratios (Table S2). The complete concordance in the sexes determined via WGS and PCR‐based methods validates the accuracy and reliability of the P8/P2 and voEP assays evaluated in this study.

**TABLE 2:** Total mapped reads, mean read depth, mean read depth ratio of sex chromosome to autosome (chr4), and mean mapping quality per chromosome for the seven individuals sequenced using whole genome sequencing.

Sample	Read count			Mean read depth			Mean read depth ratio		Mean mapping quality			Sex		
	chr4	chrZ	chrW	chr4	chrZ	chrW	Z: 4	W: 4	chr4	chrZ	chrW	WGS	P8/P2	voEP
24EP_305b	19336518	10154834	6494215	24.92	12.82	15.73	0.51	0.63	57.96	50.78	32.21	F	F	F
24EP_307b	17406719	16886573	2401736	21.59	20.39	5.86	0.94	0.27	57.82	51.25	9.25	M	M	M
24EP_307d	43229265	22843440	15701012	53.53	27.81	36.66	0.52	0.68	57.75	50.55	31.57	F	F	F
24EP_308c	19034402	10034364	6414842	25.89	13.31	16.19	0.51	0.63	58.02	50.85	32.33	F	F	F
24EP_309a	18424779	9748575	6585758	24.6	12.7	16.34	0.52	0.66	57.87	50.68	31.94	F	F	F
24EP_309b	20246983	10699508	6674229	25.82	13.36	16.04	0.52	0.62	57.99	50.75	32.11	F	F	F
24EP_309d	17134314	8998612	6015842	22.07	11.34	14.6	0.51	0.66	57.85	50.61	31.74	F	F	F

*Note:* Reads were mapped to the king penguin ( *A. patagonicus* ) reference genome (NCBI GenBank accession GCF_965638725.1), and each metric was estimated using pre‐processed data without variant quality filters.

### 3.4 Applicability of ASPCR Assay to Other Penguins

Alignment of *CHD1* sequences in six penguin species revealed that most penguins possess highly similar intron lengths (Figure 1). This suggests that sex identification based on *CHD1* introns in these penguins will produce closely associated bands that are difficult to resolve with standard gel electrophoresis. To determine whether our ASPCR assay can be used for sex assignments in other penguin species, we aligned the voEP primers to all available penguin *CHD1* sequences. The voEP_F primer is an exact match to the target region on both the *CHD1W* and *CHD1Z* sequences for all but one of the penguin species assessed (Figure 1). The *CHD1Z* sequence of gentoo penguins showed a mismatch (T/G) to this forward primer at the third nucleotide from the 5′ end. Apart from the terminal and additional mismatch added, the voEP_R primer is also fully complementary to the target region on the *CHD1W* and *CHD1Z* sequences for all penguin species. As an identical 3′ terminal mismatch (G/A) is present for all species between the reverse primer and the *CHD1Z* sequence, this primer would likely work as an allele‐specific primer for the sexing of all penguins (Figure 1).

## 4 Discussion

In this study, we developed a new primer set based on allele‐specific PCR (ASPCR) for sex identification of emperor penguins using non‐invasively collected guano. By targeting a conserved exonic region of the *CHD1* gene, we successfully designed the voEP_F/voEP_R/P2 primer set to amplify short and distinguishable fragments from *CHD1W* and *CHD1Z*. Although both intron‐based primers could be used for sexing in emperor penguins, they were less reliable when used with more challenging DNA samples. Allelic and locus dropouts were apparent with the 2550F/2718R primer set in feather samples, likely because the target amplicons are significantly larger (~650–750 bp). While the P8/P2 primer set performed better than 2550F/2718R, it remained less effective compared to the voEP assay, particularly when guano samples were used. Our results indicate that the voEP primer set is sensitive and reliable across a wide range of DNA quantity and quality and may also be used for sex identification in other Spheniscidae species.

The overall performance of the voEP assay was better than that of P8/P2 across both plucked feather and guano samples. More individuals were successfully sexed with the voEP primer set, presumably because it targets smaller PCR products that are more likely to amplify when DNA is highly degraded (Glenn et al. 1999; Hughes‐Stamm et al. 2011). Consistent with this explanation, a more pronounced difference in amplification success between the two primer sets was observed in guano samples. The voEP assay also produced bands that were easier to resolve due to the larger size difference between its *CHD1W* and *CHD1Z* products. In contrast, the P8/P2 primers showed limited band resolution, as observed in other penguin species (Costantini et al. 2008; Faux et al. 2014), because the amplicons were more similar in size. Although greater band separation can be achieved by running a longer gel or applying a higher voltage, these changes either reduce sample throughput or necessitate less conductive gels to prevent generation of excessive heat and band distortion (Singhal et al. 2010). With improved sample coverage and convenience, the voEP primer set therefore outperforms the P8/P2 primers for sexing of emperor penguins.

While the sexes determined by both P8/P2 and voEP primer sets were almost identical in plucked feather samples, their level of agreement was lower when guano was used. Sex discrepancies in the guano samples showed a unidirectional bias, where P8/P2 consistently yielded a single male band when the voEP assay yielded double bands. Given that 30.8% of these mismatched samples were also detected as females by P8/P2 in at least one technical replicate, it suggests that those samples were most likely misidentified as males due to allelic dropouts. Although dropout of the larger allele would lead to false male calls with the P8/P2 assay, it does not cause misassignment with the voEP primers because the smaller product is female‐specific. This characteristic increases the reliability of the voEP assay across various sample types. As every PCR‐based approach is susceptible to errors (Pompanon et al. 2005), it remains possible that rare point mutations, polymerase infidelity, or amplification artifacts may have led to misidentification of some discordant samples by the voEP primers. However, technical replicates of the voEP assay yielded consistent results, which reduces the likelihood of erroneous assignments due to stochastic errors (Navidi et al. 1992; Taberlet et al. 1999). Furthermore, our independent sex validation via WGS in a subset of high‐quality feather samples showed 100% congruence in the sex assignments. This observed consistency provides an empirical validation for the accuracy and reliability of our new assay.

By isolating the effects of degradation and template quantity, we found preliminary evidence that voEP primers are more sensitive compared to the P8/P2 primers. Strong amplification was consistently achieved with the voEP primer set, enabling accurate sexing even with low quantities of highly fragmented DNA. The higher sensitivity of the voEP primers at smaller DNA fragment sizes is not surprising, as the limited availability of intact DNA would have reduced or precluded the amplification of larger PCR products (Hughes‐Stamm et al. 2011; Tvedebrink et al. 2012). At larger DNA fragment sizes, however, the greater sensitivity of the voEP assay is more likely attributed to the lack of degeneracy in its primer sequence. While degenerate bases in the P8/P2 primers allow amplification of more divergent sequences, their inclusion also reduces the concentration of the perfectly matched primer, which can in turn compromise the assay sensitivity (Rose et al. 1998; Zheng et al. 2008). To maintain broad applicability across species, the voEP primers were instead designed to target a more conserved region of the *CHD1* gene.

Evaluation of *CHD1* sequences in five additional Spheniscidae species revealed that the voEP primer set may also be used for sex assignments in other penguins. Although the voEP_F primer has a mismatch to the *CHD1Z* sequence of gentoo penguins (T/G), the mismatch occurs near the 5′ end which should have a low impact on the primer extension efficiency (Wu et al. 1989). Nonetheless, this primer can easily be optimized for this species by decreasing the annealing temperature or matching the primer to the gentoo sequence. The voEP_R primer can theoretically work as an allele‐specific primer in all species assessed as the targeted C → A point mutation is present between their W and Z alleles. While it is rare, an alternative form of Z allele that possesses a C instead of the usual A nucleotide has been detected in a potentially introgressed population of Magellanic penguins (Hibbets et al. 2020). In such a case where the same target terminal base is present in both W and Z copies of the *CHD1* gene, the voEP_R primer will not be able to differentiate between males and females. Therefore, a different point mutation must be chosen to enable the sex‐typing of individuals in such populations.

In conclusion, the voEP_F/voEP_R/P2 primer set is a robust and reliable ASPCR‐based assay that can be used for sex identification in emperor penguins. Intron‐based sexing, particularly with P8/P2, can be reliably used in this species when ample amounts of high‐quality DNA are available. However, our results showed that the P8/P2 primer set is less reliable when guano samples were used, presumably because those samples are more degraded compared to plucked feathers. Our assay presented here targets smaller PCR products, making it suitable for use with a variety of sample types—including highly degraded environmental (eDNA) and ancient DNA. A major limitation in this study is that biological samples were collected from individuals of unknown sex. Without physical confirmation, we had limited ability to fully assess the accuracy of any single assay. That said, the high concordance of two distinct molecular approaches (intron‐based and allele‐based) and the independent validation via WGS provided added confidence to the accuracy of our results. Our degradation and dilution experiment also provided preliminary evidence to the increased analytical sensitivity of the voEP assay, but more replicates would be needed to generate robust sensitivity estimates. Although ASPCR‐based sexing requires that the *CHD1W* and *CHD1Z* sequences of a target species be known prior to primer design, we showed that it is an efficient and reliable alternative method for sexing in these penguins, particularly when the standard approach is unsuitable. As illustrated through sequence alignments, the voEP primer set may also be used for sex assignments in other penguin species, though empirical testing will be needed to confirm its applicability.

## Author Contributions

**Vicky Ooi:** conceptualization (lead), data curation (lead), formal analysis (lead), investigation (lead), methodology (lead), project administration (lead), validation (lead), visualization (lead), writing – original draft (lead), writing – review and editing (lead). **Sarah R. Smith:** funding acquisition (supporting), resources (equal), supervision (lead), validation (supporting), writing – review and editing (equal). **Skye Davis:** data curation (supporting), formal analysis (supporting), methodology (supporting), writing – original draft (supporting), writing – review and editing (equal). **David R. Thompson:** resources (supporting), writing – review and editing (equal). **Matt H. Pinkerton:** funding acquisition (equal), writing – review and editing (equal). **Sarah P. Flanagan:** methodology (supporting), resources (supporting), writing – review and editing (equal). **Michelle LaRue:** methodology (supporting), resources (supporting), writing – review and editing (equal). **Birgitte I. McDonald:** funding acquisition (equal), resources (equal), writing – review and editing (equal).

## Funding

Funding for fieldwork and sample collection was provided by the NSF grant #194355, the Ministry of Business, Innovation and Employment (‘Ross Sea Research and Monitoring Programme’, contract number CO1X1710), the Antarctic Science Platform Phase 1 (ANTA1801) and Phase 2 (ANTARCTICANZ2504) managed by Antarctica New Zealand, and the Royal Society Te Apārangi Rutherford Discovery Fellowship (RDF‐UOC2202). DNA extraction, polymerase chain reactions, sequencing, and analysis were supported by the budget of the Fall 2024 MS206: Molecular Techniques class at Moss Landing Marine Laboratories, San José State University.

## Ethics Statement

All samples were collected from emperor penguins under the San José State University Institutional Animal Care and Use Committee (IACUC) protocol #1070, Antarctic Convention Act Permits #2023‐003 and #2025‐003, University of Canterbury Animal Ethics permit #2024/06R, and the Antarctic Marine Living Resources Act Permit #AMLR24_R01_LaRue_K051_2425_A.

## Conflicts of Interest

The authors declare no conflicts of interest.

## Supporting information


**Table S1:** The 2500 bp codon‐optimized sequences of the synthesized *CHD1W* and *CHD1Z* fragments. Only the flanking sequences were codon‐optimized; the target regions (i.e., primer and original *CHD1* sequences; in red) were unmodified.
**Figure S1:** Gel images of all samples amplified using the 2550F/2718R primer set. The expected *CHD1W* and *CHD1Z* bands were ~750 bp and ~650 bp, respectively (Zhang et al. 2013). Five samples yielded one band with this primer set, suggesting that they may be males (M?), but the same samples were identified as females with the P8/P2 and voEP primers. Various non‐specific amplifications were present in the PCRs despite efforts made to optimize the assay. MW, molecular weight ladder, NTC, no‐template control.
**Figure S2:** Representative gel images of all feather samples amplified using the P8/P2 primer set. The amplified *CHD1Z* and *CHD1W* fragments were 368 bp and 388 bp, respectively. Full dataset with outcome of PCR replicates is available at https://github.com/vicky‐ooi/EP_Molecular_Sexing. (A–G): Feather samples collected from emperor penguins in 2022 and 2023. These PCR products were run on a 3% EtBr‐stained agarose gel in TAE buffer for 1 h 45 min at 10 V/cm. (H–I): Feather samples collected from emperor penguins in 2024. These PCR products were run on a 3% EtBr‐stained agarose gel in LAB buffer for 1 h 45 min at 20 V/cm. MW, molecular weight ladder, NTC, no‐template control.
**Figure S3:** Representative gel images of all feather samples amplified using the voEP primer set. Full dataset with outcome of PCR replicates is available at https://github.com/vicky‐ooi/EP_Molecular_Sexing. The common product for *CHD1Z* and *CHD1W* was 104 bp, and the *CHD1W*‐specific product was 74 bp. (A–G): Feather samples collected from emperor penguins in 2022 and 2023. These PCR products were run on a 3% EtBr‐stained agarose gel in TAE buffer for 1 h 30 min at 10 V/cm. (H–I): Feather samples collected from emperor penguins in 2024. These PCR products were run on a 3% EtBr‐stained agarose gel in LAB buffer for 45 min at 20 V/cm. MW, molecular weight ladder, NTC, no‐template control.
**Figure S4:** Representative gel images of all guano samples amplified using the P8/P2 primer set. Full dataset with outcome of PCR replicates is available at https://github.com/vicky‐ooi/EP_Molecular_Sexing. The amplified *CHD1Z* and *CHD1W* fragments were 368 bp and 388 bp, respectively. All PCR products were run on a 3% EtBr‐stained agarose gel in LAB buffer for 1 h 45 min at 20 V/cm. This resulted in slightly greater separation between the *CHD1Z* and *CHD1W* bands. MW, molecular weight ladder, NTC, no‐template control.
**Figure S5:** Representative gel images of all guano samples amplified using the voEP primer set. Full dataset with outcome of PCR replicates is available at https://github.com/vicky‐ooi/EP_Molecular_Sexing. The common product for *CHD1Z* and *CHD1W* was 104 bp, and the *CHD1W*‐specific product was 74 bp. All PCR products were run on a 3% EtBr‐stained agarose gel in LAB buffer for 45 min at 20 V/cm. MW, molecular weight ladder, NTC, no‐template control.
**Figure S6:** Three metrics used for sex inference in seven individuals based on whole genome sequencing. The graphs represent (A) mean mapped reads, (B) mean read depth, and (C) mean mapping quality of the Z and W chromosomes after standardization against the average of an autosome (chromosome 4). Reads were mapped to the king penguin ( *A. patagonicus* ) reference genome (NCBI RefSeq accession GCF_965638725.1). Individuals are colored by the sex inferred using the PCR‐based assays (P8/P2 and voEP), which produced identical results.
**Table S2:** Mean read depth ratios of the sex chromosomes (Z or W) to the autosome (chromosome 4) calculated for each sample using both pre‐processed and processed whole genome sequencing data. For processed data, the default quality filters applied by the package XYalign were used—i.e., a minimum variant site quality of 30, minimum genotype quality of 30, minimum variant read depth of 4, and minimum mapping quality of 20. Reads were mapped to the king penguin ( *A. patagonicus* ) reference genome (NCBI RefSeq accession GCF_965638725.1). The identified male sample is highlighted in gray.

## Data Availability

All sequence data have been uploaded to the NCBI GenBank database (Accession numbers: PV563863, PV563864). Sequences of synthetic constructs and representative gel results for all samples are provided in the Supporting Information. The dataset and analytical codes used are available at https://github.com/vicky‐ooi/EP_Molecular_Sexing.
