# Supplementary material for: A Robust Assay for Molecular Sexing of Emperor Penguins Using Degraded DNA and the Potential Applicability to Other Spheniscids
Source: Ecol Evol. 2026 Nov 5;16(11):e74654. doi: 10.1002/ece3.74654 (PMC13739418; doi:10.1002/ece3.74654)
Supplement: Supplementary file 1 [file ECE3-16-e74654-s001.docx]

**Supplemental Information for:**

**A robust assay for molecular sexing of emperor penguins using degraded DNA and the potential applicability to other Spheniscids**

Vicky Ooi^1^*, Sarah R. Smith^1^, Skye Davis^2^, David R. Thompson^3^, Matt H. Pinkerton^3^,

Sarah P. Flanagan^2^, Michelle LaRue^4^, Birgitte I. McDonald^1^

^1^Moss Landing Marine Laboratories, San José State University, Moss Landing, California, USA.

^2^School of Biological Sciences, University of Canterbury, Christchurch, New Zealand.

^3^New Zealand Institute for Earth Science, Hataitai, Wellington, New Zealand.

^4^Gateway Antarctica, School of Earth and Environment, University of Canterbury, Christchurch, New Zealand.

*Author for correspondence: Vicky Ooi (Email: vicky.ooi@sjsu.edu)

**Table of Contents:**

| **Table S1** | Pages 2 – 4 |
| --- | --- |
| **Figure S1** | Pages 4 – 5 |
| **Figure S2** | Pages 5 – 6 |
| **Figure S3** | Pages 6 – 7 |
| **Figure S4** | Page 7 |
| **Figure S5** | Page 8 |
| **Figure S6** | Page 9 |
| **Table S2** | Page 9 |

**Table S1.** The 2500 bp codon-optimized sequences of the synthesized *CHD1W* and *CHD1Z* fragments. Only the flanking sequences were codon-optimized; the target regions (i.e., primer and original *CHD1* sequences; in red) were unmodified.

| **Synthesized gene** | **Codon-optimized sequence (5**′ **to 3**′**)** |
| --- | --- |
| ***CHD1W*** | GATGCCAGTGTCAGCCAGCTCAGTATCAGCTTTTAATGAGATGTTTTCTCTCCTATTCAGGACAGCTATCATCAGTATAAGAAACTGAAGATTCACGACGTCTTTTGCTAGTGAAACAAGACCCATCTGTGATGCGTGAACGATTAGAATTCACGCTAGTTCGAACACGTGAACGACTGAAATTCTCGGTAATTTTTTGTCCTGTTCACCGCCTTTGTGGTGAAGCAGCTGTGGTATCATTAGACACAGAATATCAAGTTCGCCTTCTTGAGTAGATAGTTGCTGGAAGTTGCATTCAGGCCAGAAGTCAACCTCAACGCTCGATATTTTTAGTGCTACCAGTTGAACATTTTGTGATTTGGATGTAAGAAAAAGTGCCGCGCTTGATGCATTTGCTTTGATCAGTGAATAATAAAGTGATTGTCCTGCTGATCTTTTATATTTAACATCTTCTGATTTCTTCAGTGCATGAACGTGTTTGGTTAATTCTACATTATTGTCAGCCACTGACTCAGGCATCCGCACGTGATAAGTCCAATCTTGAAGGAAGTGAAAAAACAAATAATTCAACTTTCAGGGCGCTGTTTTTTCATGCACCTCTATTAGAGTACTTTTAAGCGCCGAAAGAGATTCAGCTCATTCCTCATTCTGGCACACGTCGCCTGTAGCTTGTGTCTGTTCCACAAGAGCAAAAATACTTTCATAAGGGATTCTGTATCTTGAAGCATTCTCTATGTGTGAATCCTGTATATCCTGTATAAAACCGTGATTTATACAAACACATGAAATATGTGAATGCTGAAAATTTGTCATCTGGACTTCTGCTTCCCAAAGATTCTGGTGGCTCAGAGCTTCTCTAATAAGGTGATGTATTAATTTGATGAAAACACTTACATCTTGATCCACAAGGTCGCAAACTTCAGCAACATGGACGAGGACGATATTGAGCTGGAGCCCGAAAGAAACTCACGGAATTGGGAAGAGATCATCCCTGAAGTGCAGAGGAGGAGGATTGAGGAGGAAGAGAGGCAGAAGGAACTTGAGGAGATTTATATGCTCCCAAGGATGAGGAACTGTGCAAAACAGGTATCTCTGGGTTTTAACCAACTAACTTTGATTTTTTTGTTGTTGTTGTTGTTTGTTTGTTTTTTCGTTGCTGTTGTTTTGGCTTGTACTTTTGGGTTGGGTGGTTTTCACGCGTGGCGCCCCCTCCCCCCATTTTTGACAGGCTAGATAACACATTAATAAAATCTTTGTCACATAGCTTTGAACTACTTAATCTGAAATTCCAGATCAGCTTTAATGGAAGTGAAGGGAAACGCAGTAGGAGCAGAAGATATTCTGGATCTGATAGTGACTCCATCTCAGAAAGAAAACGACCAAAAAAACGTGGACGACCACGAACTATTCCTCGAGAAAATATTAAAGGATTTAGCGATGCAGAGATCAAGCCGTGCGATATGGAGGGACCTGTATGTTTGAAGGAACCCGGCAGATCTATTATGACTACTATTACAGGGCGTGAAAGTATCGCAGACAGTTCATCTTCCCCGAATCTATCTTGATTTTCTTTCTGTTCTGAGCCCTGAACGTTTTGCCTTGTGTGCTTTGCGGTGCAGATATTGTGAATTGCCACGGTAAGAATCTGAATCCATTTTGATGAGGCATCAAACTGATCCTTAAAAATATCCTCTGTTGTTTCAAATTTGTCAATTACTGCCACAGCAAAAACTCCTGTTACAACGGCTAGGCCTGTTTCTAACATACTTCTAAAAATAACTTGGTGCCAATCATACTGTCTAAAAACTGCTCCGCCGAGAGAGTGTGCAAGAGCTTCTGGTGACAGCTTCAGAAATTTGAGTGGATCCTGTTCTACTTCAAGACAAATTGACACTGCAACTGAATTAACGTTATTAGTCGGTTCTTTAAACTGAAGAAATACAAAGTGTTGTATTCTAGGTACAAGATTCTGTATAGCAGATCACTGCAGTACCTCGTGCTTAAACACTTTTTCGGTAGACGCAGGAGGAATTGTTTCGTTCTCCCCTTCCTTGGGCTGTAGAATGCCACGCTCCTCTTGAAGTTCAAGTATGCTTGCTTTAGTAAAATGTTGTGAGACCTGCAGAACATCGGGTGACGGAACAATTTTAAGGACCACTTTTGTCTGTCAATCAGTTTCTAAAATTTTATCTATTTCATTTTCATCTCTTTTCATTTTTATCCAGGTTATCTGCAGTTCCTTTCTATCACATACACAATTCTGCAGCGGGTGAGCTTCCTTAGCTATTTTCCGAAGTCCTCCAACCACCTCCTGCAGATTGCCCCTTTCACTAATAAGGCATAGCGGAACGTTTGAAATCTTTGCCTCCTCTGAATTTATATTAAATTTCTGTGACTGGACACGTCTAGCTGTTGAATTTTTTGTTTTGTCAAACTCATAAAGCAATTTCTCAAATTGATCGTCCCCTCAATAGTGCTCTTTACGTCCGTCCTCAAGCGA |
| ***CHD1Z*** | TTATGAAACCGATGCCTTTCAAAGAGCACACAGTACCTCTTCTTGATTCGCTGCCTGTTCACCTATACAGGCTAATTGCCCAGCGTGTGAAAAATCAAAAATACCTGATGCATTCTCCTGATTAAGTGAGACACGCTCATTATGTGCTGATAACTGGAGTTCTGAGTCGTTTAAACCTGCTGATAGCTGGAGTTTTAAGTGGTTTTCTGCTTCATATACTGCCTCTGCTGCTAGGCTGCCGTTGTTAGCCTGAACACAAAATATAAGGTTTGCATCTCCTGATCTATGACTTCCAGGTCCTCATTTTGAGCGTAATCAAAGCTTGAATGCCAAGTTTTCCTTGTGCTGTCCGTTAAGCATATAATAATCTGGGTTCAGAAAAAAATGCAGTCTATGATGTACCTGTTTAGATCCGTGGACTATTGAATCGCAGTGCTGTTGATAGTCCATTTCCAGCATATTCTTATTTCTACGGTGTATGAGTGTTTTTGGCTGATCCTGTATTATAGCTTCACCCTCACCCAAGCAAGTACTTGTTATTGACCCTACTTCGAGGGCTCTTGAAAGACAAACCACACCGCTATTGGCGAGGTGTTCCTTCATGCGAGTGTCCTCATCAATTTTTAGAAAAAAAAAAAGATCCTGATCTTTTCCCACCTGAGCCCTTGCTGTATGCAGAGTGTGTCAGTCAGTTGAATCTAAAAGTACTTTCATTAAAGACAGTGCTTCATGAAGCACTTTGTGTGCATCAATTTCATCTATATTATTTGAAATTCCAATATCTACAAGTACATAAAATATGTCAATGCCTGAAACATGCCCTCCAGGTTTCTGTTCTCATGAAATATCTCATGTAGCGTGTTCTTTAAATAATCACACGTACTGATCTAGTAGAAATACCTGTACTCTGACACACAAGGTGGACAACTGTTTCAGTACGGTTAACGGTAGTATTGAGTGGGGACAAGGAAGAAATTCAAGAAGCTGGGTCGGAATCACTCCAGGAGCCCCGCAGCCAAAAATAGACGGGGCGGCAAAACCAAGCGGACATAACGCAACATCTATGCTCCCAAGGATGAGAAACTGTGCAAAACAGGTGTCTCTTGGTTCTGACTGACTTGTACTTTTATGTTGCTGTTGGTTTAGTTTGTTGGGGGTTGTTGTTGGGTGTTGGTTGGGGTTTTTTTTTTTCCTTTTCTGAACACATTTTTGACAGGCAACGTAAAACTTTACTGATGTTTGTCAATCACATAGCTTTGAACTACTTATTCTGAAATTCCAGATCAGCTTTAATGGAAGTGAAGGGAGGCGCAGTAGGAGCAGAAGATACTCTGGATCTGATAGTGACTCCATCTCAGAAAGAAAAAGGCCAAAAAAACGTGGAAGACCACGAACTATTCCTCGAGAAAATATTAAAGGATTTAGCGATGCAGAGATCCGCCGCTGAGACATGGGGGGCCATGCCTGTCTGAAGGGCCAAGACCGCTTCATTATGATTATCATTATCGAGCTCGAGTCTACCGAGGGGTCCCTGTTCTTCCTGAACCTCTTCTGATTCTTCTTCTATAGCAGGCACCTGATGTTTTATCTGGTCTATTTTGTTGTGCTCACATGATGAACCGTGACTGCCCGCATTTAAACACACTTTAACGAAGGATGAAACTAATTTTAGAAGATCTTTTTCGTGGTTCTGAACCTGCTGATAACAGCAACTCAAAAAACCGTCGTGACCATGGGGGAATTGGCCTTTAATATCCAGGCTAAAATCATTTGGTTCCCTTCATTCTACCTGAAAACAGCCGTTCTTAAGGAATACGTCAATCTGTTTGGCGATAACTATAAAAACCTTTCTGGGTTCTGTTTCATCCTGAAGCTTATCGACACAGTGATAGAGCTGATGTGATATCATGCCTTTTTGAATTAAAAGAACATCAAATCTTGTACCGCCGGCACAAAAAGCTGCACAGCTGACCCCTTCAACATCTGGTGCTAGAACATCTTTCTGGTAGAGGGAGGGGGCATTGTGTTGTTCTATCTTTTCTGGGTCTGCGAAATGCTGCTGTGCTTTTGAAATCTGTCCATGCACGTATCCCTGAAGTGCTGTAAGACCTGTAAAATTTTGGTCCGCGAAATCACACTGAAGATTATATTCGCTTTCCCCTTCCACTTTCGGACACTGTTCATTCTGTTCCTCTTCCATTTCATTAGTATCCGGGGTATTTCTTCCTTTTTTCCCTCCCTTACCCAGTCCTATCGCGGATACCTGAGCTGTCACATTTTCCAGAATCTGCAGATTACTTGTTGTCGGCTCCTTCTCCTTCTCATTCGGCATAACGTGATGAGCGAGATATCAGTCTATTGTAAGTCCACGCTCAACTTTTACGATTGAACACATCCGGCAGCAGAAAGCTCCGTCCTCTGAAATTGATGATCTAATTTCTAGAACTAACTGTACCTGTTGCTTTTTTACCTTCATCAGTTTTTGAACGAAGAGAACTCT |

**Figure S1.** Gel images of all samples amplified using the 2550F/2718R primer set. The expected *CHD1W* and *CHD1Z* bands were ~ 750 bp and ~650 bp, respectively (Zhang et al., 2013). Five samples yielded one band with this primer set, suggesting that they may be males (M?), but the same samples were identified as females with the P8/P2 and voEP primers. Various non-specific amplifications were present in the PCRs despite efforts made to optimize the assay. (Abbreviations: MW = molecular weight ladder, NTC = no-template control)

**Figure S2.** Representative gel images of all feather samples amplified using the P8/P2 primer set. The amplified *CHD1Z* and *CHD1W* fragments were 368 bp and 388 bp, respectively. Full dataset with outcome of PCR replicates is available at https://github.com/vicky-ooi/EP_Molecular_Sexing. **(A)-(G):** Feather samples collected from emperor penguins in 2022 and 2023. These PCR products were run on a 3% EtBr-stained agarose gel in TAE buffer for 1 h 45 min at 10 V/cm. **(H)-(I):** Feather samples collected from emperor penguins in 2024. These PCR products were run on a 3% EtBr-stained agarose gel in LAB buffer for 1 h 45 min at 20 V/cm. (Abbreviations: MW = molecular weight ladder, NTC = no-template control)

**Figure S3.** Representative gel images of all feather samples amplified using the voEP primer set. Full dataset with outcome of PCR replicates is available at https://github.com/vicky-ooi/EP_Molecular_Sexing. The common product for *CHD1Z* and *CHD1W* was 104 bp, and the *CHD1W*-specific product was 74 bp. **(A)-(G):** Feather samples collected from emperor penguins in 2022 and 2023. These PCR products were run on a 3% EtBr-stained agarose gel in TAE buffer for 1 h 30 min at 10 V/cm. **(H)-(I):** Feather samples collected from emperor penguins in 2024. These PCR products were run on a 3% EtBr-stained agarose gel in LAB buffer for 45 min at 20 V/cm. (Abbreviations: MW = molecular weight ladder, NTC = no-template control)

**Figure S4.** Representative gel images of all guano samples amplified using the P8/P2 primer set. Full dataset with outcome of PCR replicates is available at https://github.com/vicky-ooi/EP_Molecular_Sexing. The amplified *CHD1Z* and *CHD1W* fragments were 368 bp and 388 bp, respectively. All PCR products were run on a 3% EtBr-stained agarose gel in LAB buffer for 1 h 45 min at 20 V/cm. This resulted in slightly greater separation between the *CHD1Z* and *CHD1W* bands. (Abbreviations: MW = molecular weight ladder, NTC = no-template control)

**Figure S5.** Representative gel images of all guano samples amplified using the voEP primer set. Full dataset with outcome of PCR replicates is available at https://github.com/vicky-ooi/EP_Molecular_Sexing. The common product for *CHD1Z* and *CHD1W* was 104 bp, and the *CHD1W*-specific product was 74 bp. All PCR products were run on a 3% EtBr-stained agarose gel in LAB buffer for 45 min at 20V/cm. (Abbreviations: MW = molecular weight ladder, NTC = no-template control)

**Figure S6.** Three metrics used for sex inference in seven individuals based on whole genome sequencing. The graphs represent **A)** mean mapped reads, **B)** mean read depth, and **C)** mean mapping quality of the Z and W chromosomes after standardization against the average of an autosome (chromosome 4). Reads were mapped to the king penguin (*A. patagonicus*) reference genome (NCBI RefSeq accession GCF_965638725.1). Individuals are colored by the sex inferred using the PCR-based assays (P8/P2 and voEP), which produced identical results.

**Table S2.** Mean read depth ratios of the sex chromosomes (Z or W) to the autosome (chromosome 4) calculated for each sample using both pre-processed and processed whole genome sequencing data. For processed data, the default quality filters applied by the package XYalign were used – i.e., a minimum variant site quality of 30, minimum genotype quality of 30, minimum variant read depth of 4, and minimum mapping quality of 20. Reads were mapped to the king penguin (*A. patagonicus*) reference genome (NCBI RefSeq accession GCF_965638725.1). The identified male sample is highlighted in grey.

| Sample | Pre-processed | | Processed | |
| --- | --- | --- | --- | --- |
|  | **Z/4** | **W/4** | **Z/4** | **W/4** |
| 24EP_305b | 0.51 | 0.63 | 0.51 | 0.62 |
| 24EP_307b | 0.94 | 0.27 | 0.99 | 0.63 |
| 24EP_307d | 0.52 | 0.68 | 0.51 | 0.67 |
| 24EP_308c | 0.51 | 0.63 | 0.51 | 0.62 |
| 24EP_309a | 0.52 | 0.66 | 0.51 | 0.66 |
| 24EP_309b | 0.52 | 0.62 | 0.51 | 0.61 |
| 24EP_309d | 0.51 | 0.66 | 0.51 | 0.65 |
